# Simultaneous TGF-β and GITR pathway modulation promotes anti-tumor immunity in glioma

**DOI:** 10.1007/s00262-025-04098-w

**Published:** 2025-06-28

**Authors:** Daniela Lorizio, Manuela Silginer, Julia Friesen, Alan L. Epstein, Michael Weller, Patrick Roth

**Affiliations:** 1 Department of Neurology and Brain Tumor Center, University Hospital Zurich and University of Zurich, Frauenklinikstrasse 26, 8091 Zurich, Switzerland; 2 Department of Pathology, Keck School of Medicine, University of Southern California, Los Angeles, CA USA

**Keywords:** Glioblastoma, GITRL, Microenvironment, Immunotherapy, Immunosuppression

## Abstract

The immunosuppressive tumor microenvironment of glioblastoma limits the effectiveness of most immunotherapies. Transforming growth factor (TGF)-β signaling drives tumor progression and prevents effective T cell activity. Notably, both regulatory T cells (Tregs) and effector T cells within glioblastoma and other tumors express high levels of the immune checkpoint receptor, glucocorticoid-induced tumor necrosis factor receptor (GITR), which modulates T cell activation and function. Combining GITR agonism with TGF-β inhibition may therefore offer a compelling approach to restore anti-tumor immunity. We evaluated the combined effects of TGF-β inhibition and GITR modulation using two different GITR agonists in syngeneic mouse glioma models. GITR modulation enhanced T cell activation, as shown by increased cytokine secretion and effector T cell proliferation in vitro. Combining GITR modulation with TGF-β inhibition amplified these effects, resulting in significantly stronger immune cell-mediated tumor cell killing compared to single-agent treatments. Combination therapy improved survival of glioma-bearing mice, with a higher fraction of long-term survivors compared to monotherapy. Surviving mice resisted tumor re-challenge, indicating durable adaptive immunity. In summary, dual targeting of TGF-β and GITR pathways synergistically enhances anti-tumor immunity in glioblastoma. This novel combination strategy demonstrates clinical potential by addressing the limitations of existing immunotherapies and offering a promising approach for durable and effective glioblastoma treatment.

**Supplementary Information:**

The online version contains supplementary material available at 10.1007/s00262-025-04098-w.

## Introduction

Treatment of glioblastoma remains a major challenge in the field of clinical oncology. The current standard of care including surgery, radiation therapy and chemotherapy with alkylating agents has only limited activity, and tumor recurrence occurs eventually in almost all patients [1, 2]. One of the biological hallmarks of glioblastoma is the immunosuppressive tumor microenvironment, which is characterized by low infiltration of immune effector cells and polarization of macrophages and resident microglia toward a tumor-promoting phenotype [3]. The presence of immunosuppressive signals, such as activation of the programmed cell death-1 (PD-1) pathway and the release of cytokines with immune-inhibitory properties by glioma and immune cells, contributes to the malignant phenotype and poor prognosis of these tumors [4, 5]. Therefore, novel approaches for glioblastoma treatment focus on strategies aimed at reversing the profound immunosuppression to restore clinically meaningful anti-tumor immune responses. While immunotherapeutic agents have shown promising results in other cancers, no clinical benefit has been demonstrated in randomized phase 3 trials for glioblastoma patients to date [6–9].

Given the limited efficacy of single-modality immunotherapies, combined approaches targeting multiple immunomodulatory pathways may offer a promising strategy to induce robust anti-tumor immunity. Transforming growth factor (TGF)-β is a pleiotropic cytokine with a complex pro-tumorigenic role in glioblastoma, including strong immunosuppressive functions [10–13]. It is highly expressed by glioma cells, and activation of the TGF-β pathway correlates with poor patient prognosis [14, 15]. Despite these premises, TGF-β inhibition alone has not demonstrated clinical efficacy in patients with newly diagnosed or recurrent glioblastoma [16, 17]. However, incorporating TGF-β inhibition into a combined treatment regimen remains a promising strategy, given its potential to synergize with other therapies such as PD-1 inhibitors [18, 19]. Immune signaling pathways that can be co-targeted alongside TGF-β inhibition include members of the tumor necrosis factor receptor (TNFR) superfamily such as glucocorticoid-induced tumor necrosis factor receptor (GITR), CD137, and OX40 [20–22]. Modulation of the GITR pathway by receptor agonists has demonstrated anti-tumor activity in preclinical tumor models, primarily by destabilizing or reducing regulatory T cells, which constitutively express high levels of GITR [23–26]. Based on these findings, the first clinical trials with GITR agonists have been initiated. Compared to other TNF receptors, the biological role of the GITR pathway remains only partially understood, as GITR is also expressed on other immune cell subsets beyond Tregs, potentially influencing the therapeutic effects of GITR stimulation. In this study, we explored the interaction between the TGF-β and GITR pathways in glioma cells and lymphocytes. Subsequently, we investigated the effects of two different GITR agonists in combination with TGF-β inhibition in syngeneic glioma models, both in vitro and in vivo, laying the groundwork for further exploration of this novel dual immunotherapeutic strategy in glioblastoma.

## Material and methods

### Cell lines

The murine SMA-540 and SMA-560 glioma cell lines were provided by D.D. Bigner (Durham, NC). The CT2A glioma cell line was purchased from Sigma-Aldrich (Buchs, Switzerland). The GL-261 cell line was kindly provided by the National Cancer Institute (Frederick, MD). The cells were cultured at 37 °C and 5% CO_2_ in Dulbecco’s modified Eagle medium (DMEM) supplemented with 10% heat-inactivated fetal calf serum (FCS) and 2 mM L-glutamine (Gibco, Life Technologies Limited, Paisley, UK). Cells were regularly tested for mycoplasma infection.

## Reagents

Human recombinant TGF-β_2_ (hr-TGF-β_2_) was purchased from R&D system (Minneapolis, MN). TGF-βRI inhibitors SD208 and LY2157299 (LY) were supplied by Scios Inc. (Freemont, CA) and Eli Lilly & Co. (Indianapolis, MN), respectively. Bristol-Myers Squibb provided the following antibodies targeting the corresponding immune checkpoint receptors: anti-GITR (IgG2a, 7-mg2a clone 2D5), anti-OX40 (mIgG1 1F1RAS), anti-CD137 (mIgG1 02022017), anti-LAG3 (mIgG14H8), and anti-PD-1 (mIgG1 4H2) (BMS, Baltimore, MD). The GITRL-Fc fusion protein was generated as described [27].

## Reverse Transcriptase-quantitative polymerase chain reaction (RT-qPCR)

mRNA was isolated from cells and retro-transcribed to cDNA [28]. RT-qPCR was performed with the PowerUp SYBR green master mix (Applied Biosystems, Zug, Switzerland) and the following mouse primer pairs in the 7300 Real-time PCR System (Applied Biosystems):

HPRT1 forward: 5’-TTGCTGACCTGCTGGATTAC-3’ and reverse: 5’-TTTATGTCCCCCGTTGACTG-3’; GITR forward: 5’-GGGCCATGCTGTATGGAGTC-3’ and reverse: 5’-CCTCCTTGCCTGGAGCATACA-3’; GITRL forward: 5’-GGCTCTTGTGCATAGTGGCT-3’ and reverse: 5’-CGATGGCAGTTGGCTTGAGT-3’; CD137 forward: 5’-CTCAGCACAGAGAGCTGACA-3’ and reverse: 5’-GCACAGGACACCAAAGGTGA-3’; CD137L forward: 5’-ACACTACACAACAGGGCTCTC-3’ and reverse: 5’-GTCTTCTTCGTACCTCAGACCT-3’; OX40 forward: 5’-ATGTCATCCGTGTGAGACTGG-3’ and reverse: 5’-CCACTTCGATGGTTGCACTGT-3’; OX40L forward: 5’-GTGGTCTCTGGGATCAAGGG-3’ and reverse: 5’-TCACATCTGGTAACTGCTCCTC-3’; LAG3 forward: 5’-CCAGGCCTCGATGATTGCTA-3’ and reverse: 5’-ACGCGGTGAGTTGTAGACAG-3’; Galectin-9 forward: 5’-AAGGGGCGCAAACAGAAAAC-3’ and reverse: 5’-GGAGGGATTCCAGGAGTAGAGA-3’; PD-1 forward: 5’-TCATGAGTGCCCTAGTGGGTA-3’ and reverse: 5’-CTCCTCCTTCAGAGTGTCGTC-3’; PD-L1 forward 5’-CTCCTCGCCTGCAGATAGTT-3’ and reverse 5’-ATCGTGACGTTGCTGCCATA-3’ (Microsynth, Balgach, Switzerland). HPRT1 was used as house-keeping gene for relative quantification.

## Splenocyte isolation

Healthy donor mice were killed for spleen isolation. The organ was mashed through a 40-μm cell strainer, and a hypotonic solution was used to remove red blood cells. Splenocytes were cultured at 10^6^ cells/ml density in RPMI medium supplemented with 10% FCS, 2 mM glutamine, 1% penicillin/streptomycin (Sigma-Aldrich), and β-mercaptoethanol 1:1000 (Gibco). Anti-CD3 (1 μg/ml) and anti-CD28 (5 μg/ml) antibodies (LubioScience, Zurich, Switzerland) were added for 24 h for splenocyte stimulation. Subsequently, cells were resuspended at the same density in fresh complete RPMI containing 25 IU/ml human recombinant interleukin-2 (hr-IL-2, Peprotech, Rocky Hill, NJ) and β-mercaptoethanol.

## Flow cytometry

Cells were stained for 30 min in the dark at 4 °C for the following anti-mouse antibodies: BV421-conjugated rat anti-CD137 (BD Bioscience, Allschwil, Switzerland, 1:100), BV421-conjugated rat anti-GITR (BD Bioscience, 1:200), BV421-conjugated rat anti-PD-L1 (BD Bioscience, 1:50), APC-conjugated rat anti-PD-1 (Biolegend, San Diego, CA, 1:50), BV421-conjugated rat anti-LAG3 (BD Bioscience, 1:50), PE-conjugated rat anti-CD3 (Biolegend, 1:100), PE-conjugated rat anti-GITRL (BD Biosciences, 1:50), zombie NIR viability dye (Biolegend, 1:300). For intracellular cytokine staining, lymphocytes were incubated with GolgiPlug/GolgiStop (1:1000) (BD Biosciences) for 4–6 h at 37 °C. Subsequently, cells were incubated with CD16/CD32 Fc Block (BD Biosciences) and with the Zombie NIR dye. Staining for the following cell surface markers was performed for 30 min at 4 °C in the dark: pacific blue-conjugated anti-mouse CD4 (Biolegend, 1:100), APC-conjugated anti-mouse CD8a (Biolegend, 1:100). Fixation/Permeabilization Kit (BD Biosciences) was used prior to the following intracellular stainings: PE-conjugated anti-mouse IFN-γ (Biolegend, 1:200) and PerCP-Cy5.5-conjugated anti-mouse IL-2 (Biolegend, 1:25).

For nuclear transcription factor staining, surface markers were first stained with pacific blue-conjugated anti-mouse CD4 and APC-conjugated anti-mouse CD25 (Biolegend, 1:50). Subsequently, cells were permeabilized with FoxP3/Transcription factor staining buffer set (eBiosciences, San Diego, CA) prior to FoxP3 staining (PE-conjugated anti-mouse FoxP3, Biolegend, 1:50). Cells isolated from mouse brains from in vivo experiments were incubated with the following anti-mouse antibodies for 30 min at 4 °C in the dark: PerCp-cy5.5-conjugated anti-CD3 (Biolegend, 1:100), BV650-conjugated anti-CD4 (Biolegend, 1:50), APC-Cy7-conjugated anti-CD11b, PE-conjugated anti-CD45 (Biolegend, 1:100), BV786-conjugated anti-CD8a (Biolegend, 1:100), APC-conjugated anti-NKp46 (Biolegend, 1:100), zombie aqua viability dye (Biolegend, 1:300). Flow cytometric analyses were performed with a FACSVerse Analyzer (BD Biosciences), and FlowJo software was used for data analysis.

## Immune cell lysis assays

Target glioma cells were harvested with accutase and incubated with PKH-26 dye for 5 min (Sigma-Aldrich, 1:500). Co-culture with effector cells was set up in triplicates for 24 h at the effector:target (E:T) ratios reported in the figure legends. Live/dead cell staining was performed to assess target cell lysis by flow cytometry [29]. Immune cell-mediated tumor cell killing was assessed as the percentage of dead cells within the PKH-26-positive target cells, normalized for the background lysis (target cells in culture without effector cells).

## Immunohistochemistry

Cryosections from mouse brains were treated according to the following steps: fixation with 4% formalin, incubation with 3% H_2_O_2,_ and subsequent blocking with the Super Block solution (ScyTek Laboratories, West Logan, UT). The following primary antibodies were used: rat anti-CD45 (Biolegend, 1:1000), rat anti-CD3 (BD Bioscience), rabbit anti-CD11b (BD Bioscience, 1:500). The staining was performed at 4 °C overnight. Subsequently, the secondary antibodies Histofine Mouse MAX PO anti-rat (Biosystems, Muttenz, Switzerland) or HRP-conjugated goat anti-rat (Thermo Scientific, Waltham, MA) were added for 30 min [30]. The final staining was detected by ImmPACT DAB staining (Vector Laboratories, Burlingame, CA). Hematoxylin staining was finally performed for nuclei detection. Pictures from three tumors/group and three areas per each tumor were acquired for the different stainings and subsequently quantified. Hematoxylin-stained sections were used for tumor volumetry [31].

## Animal studies

All animal experiments were performed in conformity with the Swiss laws on animal protection as well as the ARRIVE (Animal Research: Reporting of In Vivo Experiments) guidelines and authorized by the Veterinary Office of the Canton of Zurich (approval no. ZH042/2022). C57BL/6 mice were purchased from Janvier Labs (Le Genest-Saint-Isle, France). VM/Dk mice were bred in pathogen-free facilities at the University of Zurich. Twenty thousand (20,000) SMA-560 glioma cells or 80,000 CT2A cells were stereotactically injected in the right hemisphere of four- to six-week old syngeneic VM/Dk or C57BL/6 mice, respectively. From day 5 upon tumor implantation, mice were treated according to the following schedule: the TGF-βRI inhibitor LY2157299 (daily oral gavage, 150 mg/kg weight) or solvent control (1% hydroxyethyl-cellulose HEC, 0.25% Tween 80, 0.05% Antifoam 1510-US in distilled water), anti-GITR antibody (3 mg/kg, intraperitoneal injection at day 5, 7, 9), GITRL-Fc (30 µg/dose, i.p., consecutive administrations from day 5 to day 9), or combinations thereof. The mice were observed daily according to the Cantonal Veterinary Office Zurich guidelines. Three randomized mice per group were euthanized when the first mouse in the experiment became symptomatic for further flow cytometric analysis or immunohistochemical stainings. For survival analysis, mice were euthanized when developing neurological symptoms.

## Statistics

All data were collected from at least two independent biological experimental replicates. The in vitro data are reported as mean and standard deviation (SD). T tests or one-way ANOVA testing was performed using Prism (GraphPad, Boston, MA). Kaplan–Meier curves were used for survival data in in vivo studies (with log-rank test for statistical significance *p* < 0.05).

## Results

### Immune checkpoint profiling reveals TGF-β-dependent regulation of GITR in syngeneic mouse glioma models in vitro

**Fig. 1:**
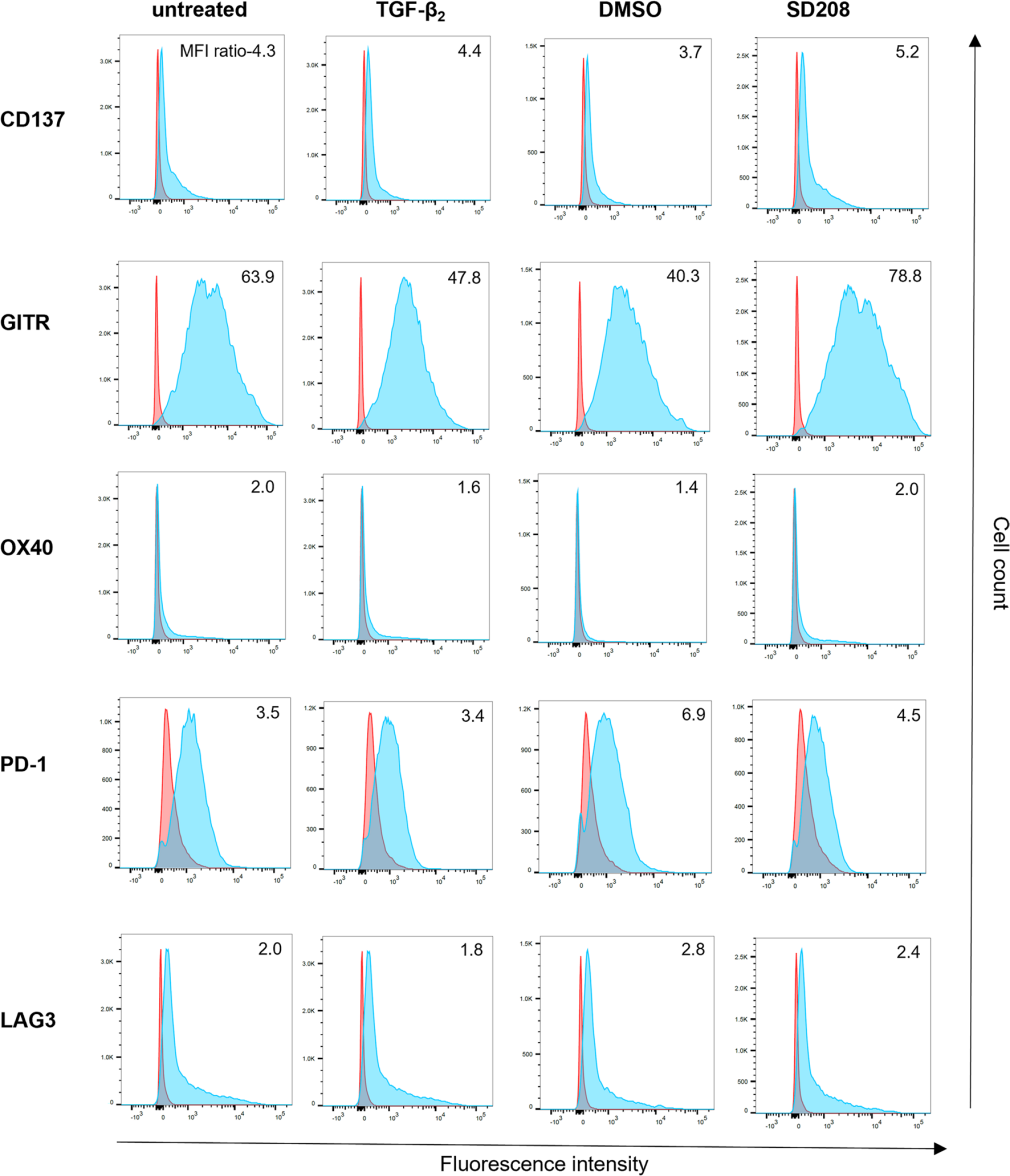
TGF-β stimulation and inhibition modulate immune checkpoint receptor expression on splenocytes*.* Splenocytes were exposed to hr-TGF-β_2_ (10 ng/ml), SD208 (1 µM), DMSO (control), or left untreated for 48 h, and immune checkpoint receptor expression was assessed by flow cytometry. Ratios of the median fluorescence intensity (MFI) of the specific antibody (blue) to its corresponding isotype control antibody (red) are shown in all panels

We first characterized the landscape of immune checkpoint receptors and the corresponding ligands in four mouse glioma cell lines (SMA-560, SMA-540, CT2A, GL-261) as well as C57BL/6-derived splenocytes (Suppl. Table 1). We observed heterogeneous transcriptional levels of PD-L1, CD137L, OX40L, and LAG3 in the mouse glioma cell lines (Suppl. Figure 1A). Upon exposure to TGF-β or the TGF-βRI inhibitor SD208, the expression levels of these molecules were not significantly altered. GITRL transcript was significantly reduced by TGF-β inhibition across the four glioma cell lines, but no effect was observed for the protein, whose surface expression was limited to only a few cells (Suppl. Figure 1B,C). Splenocytes expressed PD-1, PD-L1, OX40, LAG3, CD137, and GITR at the transcriptional and protein levels to a different extent. OX40L and GITRL transcripts were not detected in these cells. Notably, GITR protein levels were downregulated upon exogenous TGF-β exposure and upregulated following TGF-β inhibition (Fig. 1, Suppl. Figure 2A).

## Enhanced T cell activation through combined TGF-β inhibition and GITR agonism in glioma models

**Fig. 2:**
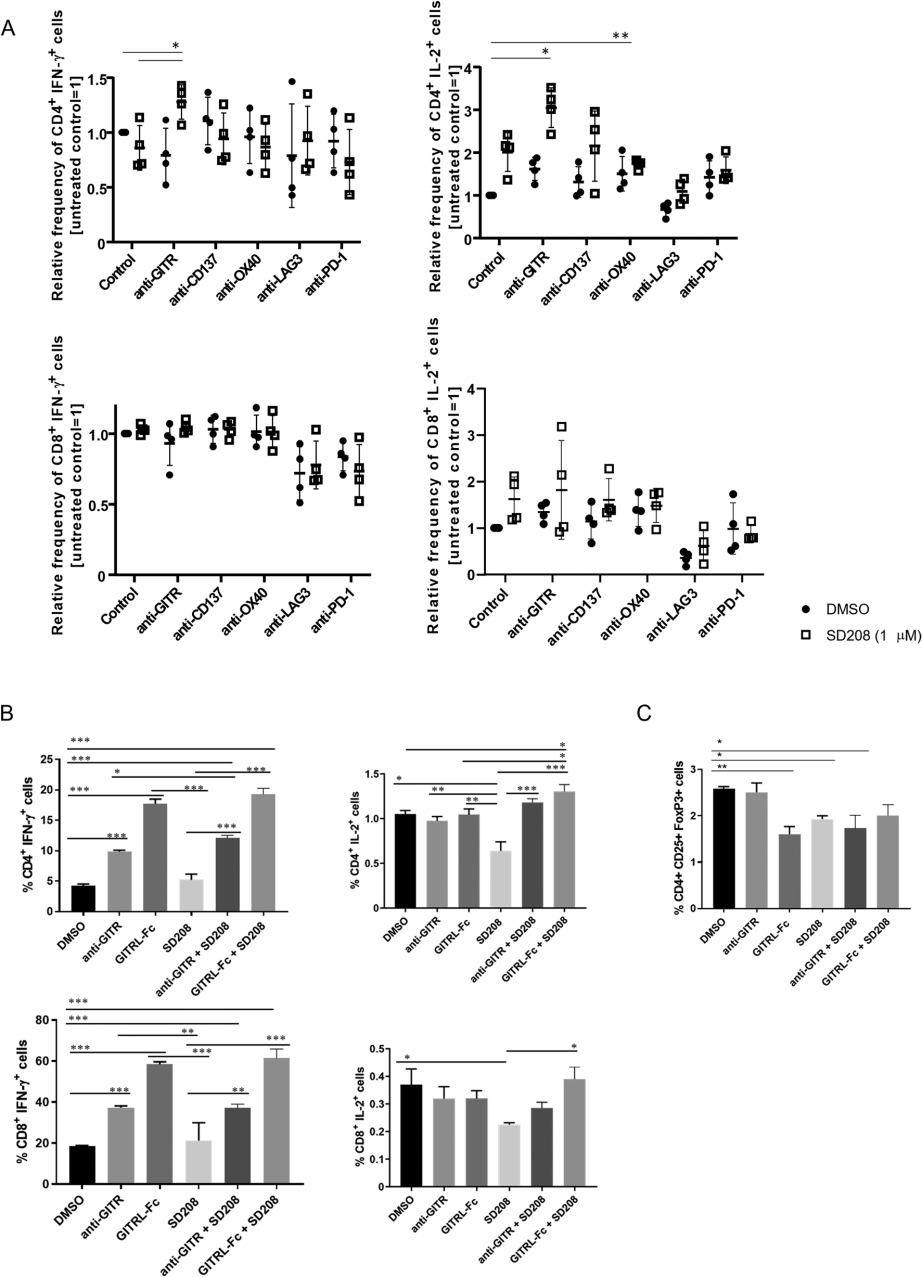
Simultaneous targeting of TGF-β and immune checkpoint receptors enhances T cell activation in syngeneic glioma co-cultures. **A**. SMA-560, SMA-540, GL-261, or CT2A glioma cells were co-cultured with syngeneic VM/Dk or C57BL/6-derived polyclonally activated splenocytes and exposed to a TGF-βRI inhibitor (SD208, 1 µM) (open symbols) or not (filled symbols), and antibodies targeting the immune checkpoint receptors GITR, CD137, OX40, LAG3, or PD-1 (10 µg/ml) or combinations thereof for 24 h (E:T = 10:1). Intracellular IFN-γ and IL-2 production by splenocytes was detected by flow cytometry, distinguishing CD4⁺ and CD8⁺ T cell subsets. For each condition, data are shown as individual values representing the four glioma cell lines. Results are expressed as mean relative frequency ± SD and were normalized to untreated control conditions (set to 1). **B**. SMA-560 cells were co-cultured with syngeneic splenocytes and exposed to an anti-GITR antibody or GITRL-Fc (10 µg/ml), either alone or combined with SD208 (1 µM) for 24 h. The percentage of CD4⁺ and CD8⁺ T cells expressing intracellular IFN-γ and IL-2 is shown for each treatment. Data are expressed as mean ± SD. C. Co-cultures as in (B) were incubated for 48 h. The Treg cell population was identified by flow cytometry as CD4^+^ CD25^+^ FoxP3^+^ cells. Data are expressed as mean ± SD. Statistical significance was assessed via one-way ANOVA with Dunnett’s post hoc test for multiple comparisons (* *p* < 0.05; ** *p* < 0.01; *** *p* < 0.001)

To assess the effect of combined TGF-β inhibition and immune checkpoint modulation on immune cell activation, we performed co-culture assays using mouse glioma cells and syngeneic splenocytes, with or without the TGF-β inhibitor SD208, antibodies targeting GITR, CD137, OX40, LAG3, or PD-1, or their respective combinations. IFN-γ and IL-2 production in CD4^+^ and CD8^+^ T cells was measured as indicators of lymphocyte activation. GITR agonism in combination with pharmacological TGF-β inhibition resulted in increased CD4^+^ T cell activation, in contrast with the limited effect observed with either treatment alone (Fig. 2A). To corroborate these findings, we performed co-culture experiments using a second GITR agonist, a murine GITRL-Fc fusion protein. This fusion protein was created by attaching murine GITRL to an IgG Fc domain, thereby mimicking the natural interaction between GITR and its ligand, which activates GITR downstream signaling [27, 32]. Compared to the anti-GITR antibody, exposure to GITRL-Fc resulted in even higher expression of IFN-γ and IL-2. Additionally, we observed that both GITR agonists, when combined with TGF-β inhibition, led to increased T cell activation compared to either single treatment or the control (Fig. 2B). Since GITR modulation may affect the Treg population, we analyzed Treg cells in the context of GITR modulation and TGF-β inhibition within the same co-culture system. Exposure to GITRL-Fc or SD208 reduced the CD25⁺ CD4⁺ FoxP3⁺ cell population, with both treatments yielding a similar effect when combined (Fig. 2C).

## Co-targeting of TGF-β and GITR signaling promotes T cell- and NK cell-mediated killing of syngeneic glioma cells

**Fig. 3:**
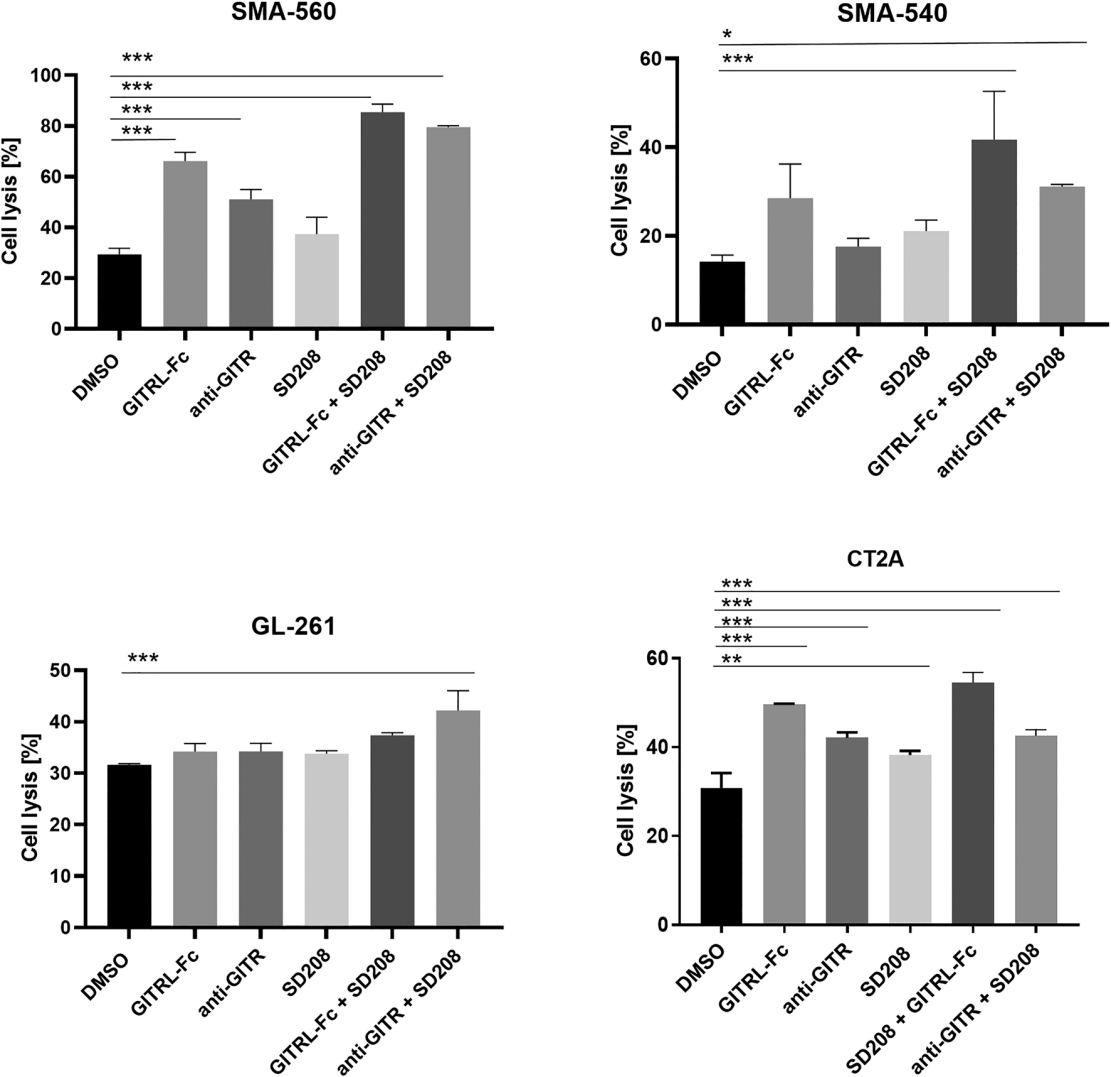
Combined TGF-β inhibition and GITR agonism enhance T cell-mediated glioma cell killing. Polyclonally activated splenocytes were exposed to an agonistic anti-GITR antibody or the GITRL-Fc fusion protein (10 µg/ml), a TGF-βRI inhibitor (SD208, 1 µM), or their combination for 48 h. Syngeneic mouse glioma cells, pre-stained with the fluorescent dye PKH-26, were co-cultured with these splenocytes for 24 h (E:T ratio = 5:1 for SMA-560 and CT2A cells, 10:1 for SMA-540, and GL-261 cells). Tumor cell lysis was assessed by flow cytometry, with the percentage of dead glioma cells shown. Data are indicated as mean ± SD. Statistical significance was determined via one-way ANOVA with Tukey’s post hoc test for multiple comparisons (* *p* < 0.05; ** *p* < 0.01; *** *p* < 0.001)

**Fig. 4:**
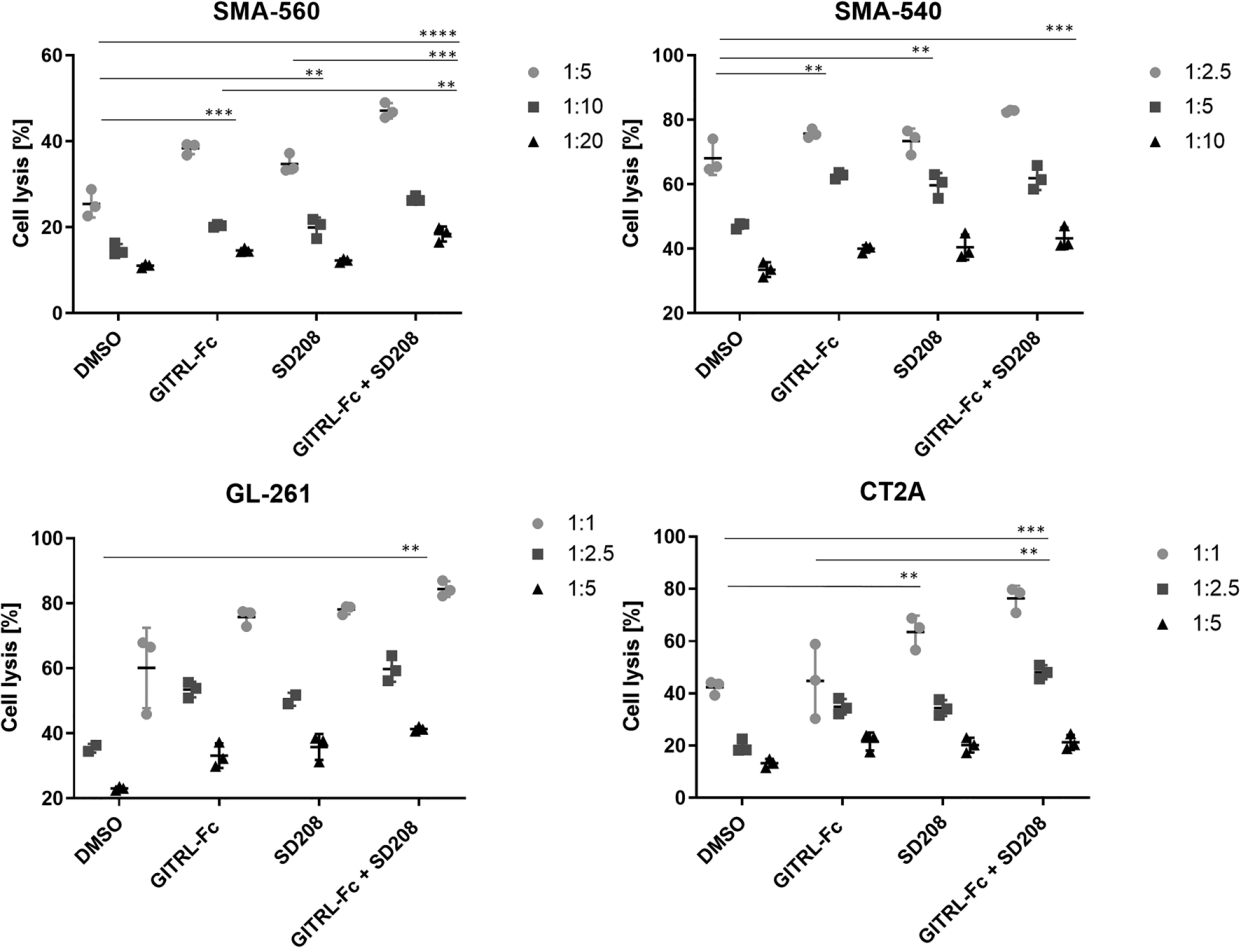
Combined inhibition of TGF-β and activation of GITR signaling increase NK cell-mediated glioma cell killing. Murine NK cells were exposed to the GITRL-Fc fusion protein (10 μg/ml), a TGF-βRI inhibitor (SD208, 1 μM), or the combination thereof for 24 h. Subsequently, PKH-26-labeled mouse glioma cells were co-cultured with the NK cells at the indicated effector:target ratios. Tumor cell lysis was assessed by flow cytometry after 20 h, as shown in the histograms. Data are presented as mean ± SD. Statistical significance was determined by one-way ANOVA with Tukey’s post hoc test for multiple comparisons (* *p* < 0.05; ** *p* < 0.01; *** *p* < 0.001)

To define the functional impact of TGF-β and GITR pathway modulation on anti-glioma immune reactivity, we exposed splenocytes to SD208, one of two GITR agonists, or the combination of both, and assessed their lytic activity against glioma cells in co-culture assays. Across all glioma cell lines, co-targeting the TGF-β/GITR pathways resulted in increased immune cell-mediated tumor cell killing compared to either treatment alone or control conditions (Fig. 3). Next, we examined the impact of TGF-β and GITR pathway modulation on the cytolytic activity of NK cells, another GITR-expressing effector immune cell subset (Suppl. Figure 2B) involved in tumor immune surveillance. Co-culture experiments involving mouse glioma cells and syngeneic NK cells at various effector:target ratios revealed that TGF-β inhibition or GITR agonism alone had limited effects on NK cell-mediated tumor cell killing. However, combined targeting of these two pathways significantly enhanced NK cell-mediated glioma cell killing compared to either treatment alone or control conditions (Fig. 4).

## Combination of TGF-β inhibition and GITR agonism prolongs survival and induces adaptive anti-tumor immunity in glioma-bearing mice

**Fig. 5:**
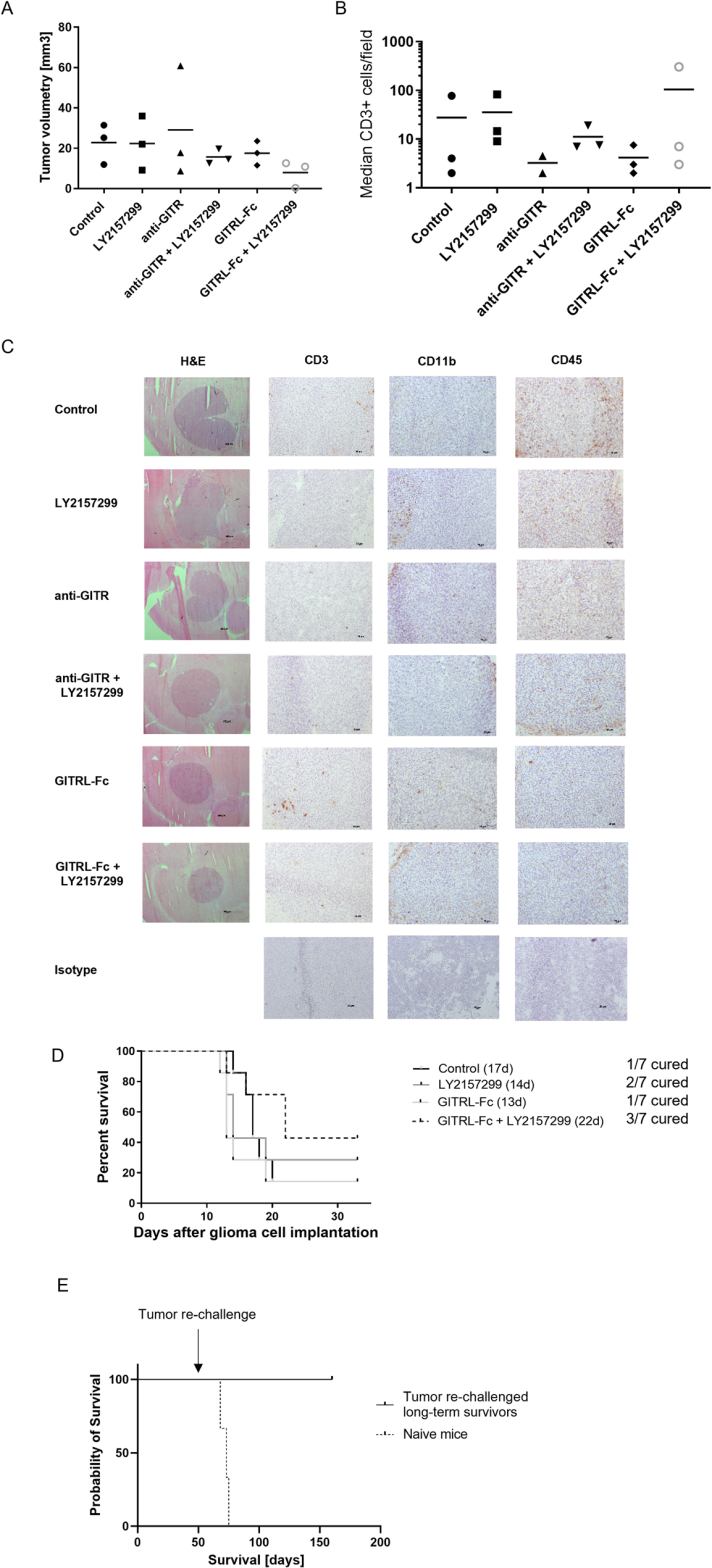
Co-targeting of TGF-β and GITR signaling in the syngeneic SMA-560 mouse glioma model increases the number of long-term surviving animals*.* **A**-**C**. Twenty thousand SMA-560 cells were intracranially inoculated into the right striatum of syngeneic VM/Dk mice. From day 5 onward, mice were treated with a TGF-βRI inhibitor (LY2157299, 150 mg/kg, daily oral gavage), an agonistic GITR antibody (3 mg/kg, i.p. at days 5, 7, and 9), GITRL-Fc (30 μg/dose, i.p. from day 5 to day 9), or the respective combinations. A. Tumor size was assessed by H&E staining of tumors from three pre-randomized mice per group, harvested when the first mouse in any group displayed neurological symptoms. The histogram shows individual tumor volumes, with bars representing the mean volume for each treatment group. **B**. CD3 immunohistochemical staining was performed on one brain section per mouse. The histogram shows the mean number of CD3 + cells in three different regions of interest from the same section. The black bar represents the mean value for each group. C. Representative images of H&E, CD3, CD11b, and CD45 staining on tumor brain sections from one mouse per group. Scale bars: 100 µm for H&E, 20 µm for other stains. D. Twenty thousand SMA-560 cells were intracranially inoculated into the right striatum of syngeneic VM/Dk mice. From day 5 onward, mice were treated with a TGF-βRI inhibitor (LY2157299, 150 mg/kg, daily oral gavage), GITRL-Fc (30 μg/dose, i.p. from day 5 to day 9), or both. Survival data are shown, including the number of long-term surviving mice. E. On day 50 post-tumor inoculation, long-term surviving mice from experiments in (D) and Suppl. Figure 3B were re-challenged with a second tumor inoculation in the contralateral hemisphere. Three naïve mice injected with tumor cells served as controls. Kaplan–Meier survival curves are shown

**Fig. 6:**
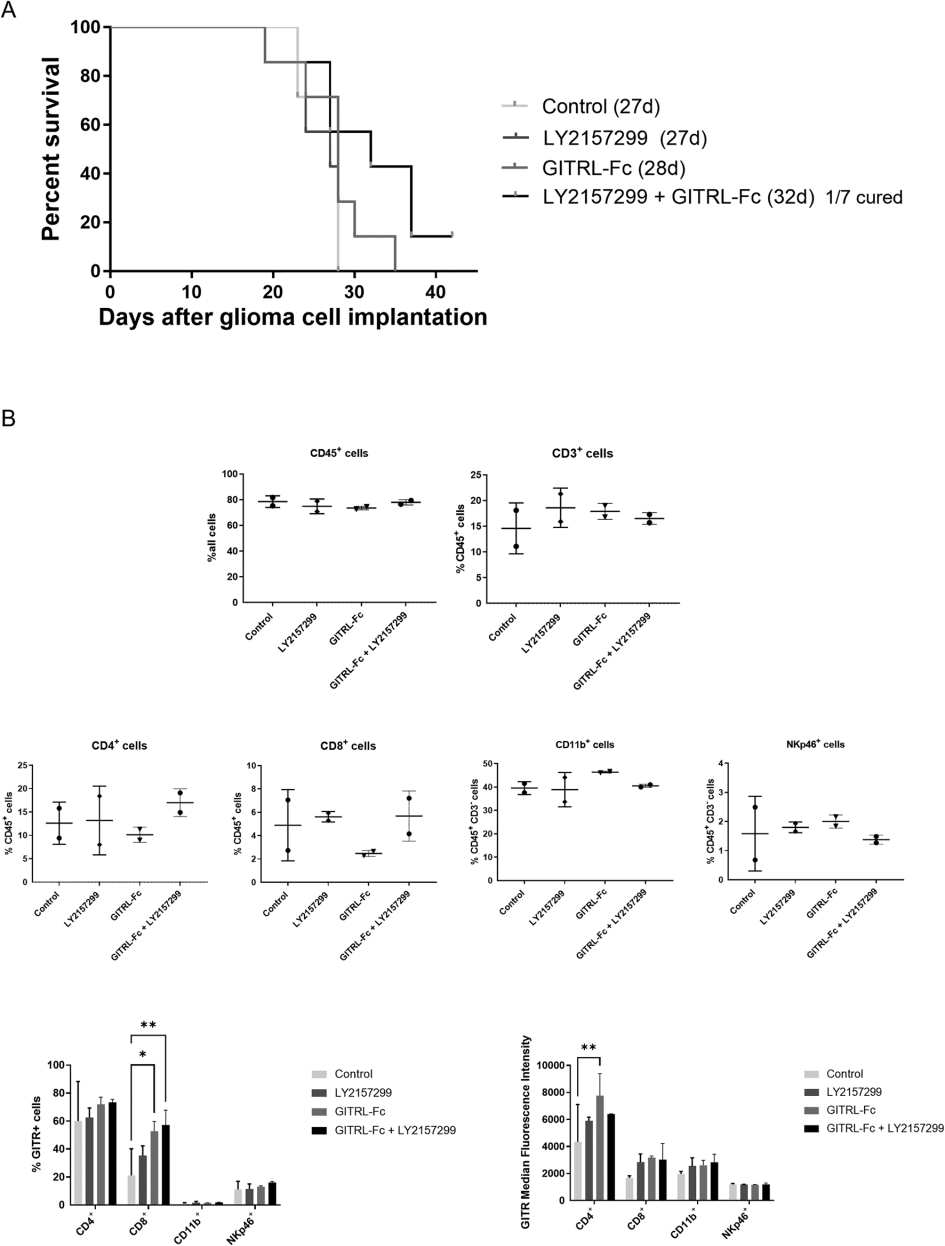
Dual targeting of TGF-β and GITR signaling improves survival and modulates the phenotype of tumor-infiltrating immune cells in glioma-bearing mice*.* **A**, **B**. CT2A glioma cells were intracranially injected into the right striatum of C57B/6 mice. Five days after tumor cell inoculation, mice were treated with a TGF-βRI inhibitor (LY2157299, 150 mg/kg, daily oral gavage), GITRL-Fc (30 μg/dose, i.p. from day 5 to day 9), or the combination. A. Survival is shown by Kaplan–Meier curves. B. Tumor-infiltrating immune cells were isolated from randomized early-stage glioma-bearing mice and analyzed by flow cytometry. Immune cell subsets were defined with the indicated cell surface markers. Each dot represents one tumor sample, and the black bars indicate the average within the same treatment group (upper panel). The lower panels show the fraction of GITR^+^ cells among these immune cell subsets and the median fluorescence intensity of GITR

To characterize GITR expression in vivo, we isolated tumor-infiltrating lymphocytes from intracranially implanted gliomas growing in syngeneic mice. Flow cytometric analysis revealed abundant GITR expression on various immune cell subsets within the tumor microenvironment, with the highest levels observed on CD4^+^ T cells and NK cells (Suppl. Figure 3A). Based on these findings and the promising in vitro data, we investigated the therapeutic effect of TGF-β and GITR pathway modulation in clinically relevant mouse glioma models. SMA-560 glioma-bearing mice were treated with either of the two GITR agonists, a TGF-β receptor inhibitor, or the combination. Combined targeting of the TGF-β and GITR pathways resulted in a trend toward tumor size reduction (Fig. 5A). The number of tumor-infiltrating CD45^+^ immune cells, CD3^+^ T cells, or CD11b^+^ myeloid cells was similar across the treatment groups (Fig. 5B,C). Treatment with the TGF-β receptor inhibitor, anti-GITR, or GITRL-Fc alone did not prolong the survival of glioma-bearing mice, whereas the combined modulation of the two immune checkpoints led to a higher fraction of long-term surviving mice (Fig. 5D, Suppl. Figure 3B). These long-term survivors were protected from a second tumor cell inoculation in the contralateral brain hemisphere, suggesting that an adaptive anti-tumor immune response was induced by the therapeutic intervention (Fig. 5E). To confirm these results in a second glioma mouse model, we treated CT2A glioma-bearing mice with the same approach. Again, there was a trend for longer survival (32 vs 27 days) and one long-term surviving mouse in the combinatorial treatment arm (Fig. 6A). Ex vivo analyses of explanted tumors showed a similar distribution of immune cells across the four groups. A detailed characterization revealed a higher fraction of GITR-expressing CD8^+^ T cells in mice treated with a TGF-β receptor inhibitor in combination with GITRL-Fc than in control mice. Furthermore, GITR expression on intratumoral CD4^+^ T cells was highest in mice receiving GITRL-Fc, suggesting a feedback loop in the GITR expression/pathway activation in syngeneic gliomas (Fig. 6B).

## Discussion

Several immunotherapies have shown striking results in cancer treatment, but none have proven effective against glioblastoma to date. One contributing factor is the expression of soluble and membrane-bound immunosuppressive molecules by glioma cells and by their microenvironment, which prevents the induction of effective anti-tumor immune responses [33].

Here, we first characterized the expression landscape of a panel of immune stimulatory and immunosuppressive checkpoint molecules in murine glioma cell lines as well as in syngeneic immune cells and explored the potential impact of a key immunosuppressive mediator, TGF-β, on the expression of these molecules. TGF-β signaling drives key biological hallmarks of glioblastoma, including invasiveness and tumor-derived immunosuppression making it a central biological hub [34]. Therefore, it has traditionally been regarded a promising target for a therapeutic intervention. We observed that most immune checkpoint ligands and receptors were expressed by both glioma cells and immune cells (Fig. 1, Suppl. Figure 1). TGF-β stimulation reduced the levels of immune stimulatory GITR, while TGF-β inhibition increased GITR expression in lymphocytes, suggesting an interaction between the two pathways. Previous studies have shown downregulation of immune-stimulating pathways, such as NKG2D signaling, by TGF-β [35]. Based on these findings, we hypothesized that impaired GITR signaling may contribute to the immunosuppressive network established by TGF-β. In line with this assumption, TGF-β also had an inhibitory effect on the expression of GITRL at the transcriptional level, but protein levels were below the detection limit (Suppl. Figure 1). GITR plays a co-stimulatory role in cancer immunity, but, unlike other TNFR family members, its function across various immune cell subsets remains incompletely understood [36]. The functional characterization of the GITR-TGF-β network in co-culture experiments using glioma cells with syngeneic immune cells revealed that exposure to an agonistic GITR antibody in combination with a TGF-β inhibitor resulted in increased T cell activation, dominantly in the CD4^+^ T cell compartment (Fig. 2A). This finding was confirmed using a GITRL-Fc fusion protein that also acts as a GITR agonist [32]. Exposure to GITRL-Fc significantly enhanced IFN-γ and IL-2 production in splenocytes, outperforming the anti-GITR antibody and suggesting superior activity of the GITRL-Fc fusion protein. The combination of any of these two GITR agonists with a TGF-β receptor inhibitor resulted in increased T cell activation (Fig. 2B). High constitutive expression of GITR has been reported on Treg cells, and GITR stimulation exerts various effects on this immune cell population [37, 38]. In preclinical cancer models, treatment with GITR agonists led to a reduction of intratumoral Treg cells or altered their immunosuppressive phenotype [24, 39–43]. Furthermore, TGF-β signaling promotes Treg differentiation and expansion [44]. Therefore, we characterized the effect of concurrent GITR agonism and TGF-β inhibition on Treg cells in co-culture experiments. Treatment with either a TGF-β inhibitor or GITRL-Fc reduced the Treg fraction, whereas the combination had no additive effect, suggesting that the combinatorial treatment may activate T cells through mechanisms beyond Treg reduction (Fig. 2C). Since the dual targeting of the TGF-β and GITR pathway increased T cell activity, we assessed its impact on T cell-mediated tumor cell killing in vitro. TGF-β inhibition in combination with either anti-GITR or GITRL-Fc resulted in enhanced T cell-mediated lysis of glioma cells (Fig. 3). Previous studies have shown that TGF-β signaling reduces NK cell activation, whereas the effect of GITR stimulation on the NK cell compartment has yielded conflicting data [45–47]. Since NK cells may play a role in glioma immunosurveillance [48], we investigated the effects of combined TGF-β and GITR modulation on their activity. Compared to single-pathway modulation, the dual treatment significantly enhanced NK cell-mediated tumor cell killing (Fig. 4). These results suggest that GITR stimulation affects not only T cell-mediated immune responses but also innate immune cells.

Ex vivo analyses confirmed high GITR expression in the tumor microenvironment of glioma-bearing mice (Suppl. Figure 3A). Therefore, we explored the effect of single or dual targeting of TGF-β and GITR in immunocompetent syngeneic mouse glioma models. In SMA-560 glioma-bearing mice, the tumor size at an early stage was similar across the different treatment groups, except for a trend in volume reduction for animals treated with GITRL-Fc and a TGF-β receptor inhibitor (Fig. 5A). The difference observed between the two GITR agonists may be explained by a stronger immune stimulation obtained with the GITRL-Fc fusion protein, as observed in the in vitro experiments. No significant variation in infiltrating immune cells at early time points was detectable by immunohistochemical staining among the treatment groups (Fig. 5B, C). In some preclinical cancer models, TGF-β inhibition has been observed to reverse T cell exclusion [49, 50]. However, consistent with previous findings in experimental gliomas [51], we did not observe an increase in the limited intratumoral T cell infiltration following TGF-β inhibition. This may be due to other immunosuppressive mechanisms that counteract the effects of TGF-β inhibition. Combined treatment with a TGF-β inhibitor and either anti-GITR antibody or GITRL-Fc resulted in a trend toward prolonged survival and an increased number of long-term survivors (Fig. 5D, Suppl. Figure 3B). The variation in tumor volumes and immune cell infiltration across treatment groups may explain the survival data, with some mice being cured and others showing no treatment benefit. These preclinical data align with the effects of established cancer immunotherapies, which typically yield a fraction of long-lasting responders and a larger proportion of non-responders [52]. Similar results were obtained in a second syngeneic glioma model, where the combinatorial treatment did not significantly impact median survival overall, while long-term survivors were observed again (Fig. 6A). Flow cytometry analysis of dissected brains from early-stage CT2A glioma-bearing mice revealed no substantial differences in immune cell infiltration across treatment groups. The high fraction of CD11b^+^ myeloid cells and low number of NK cells in the tumor microenvironment may represent a key barrier to improved therapeutic efficacy. Targeting the myeloid compartment could therefore be a promising strategy to enhance immune responses and further improve therapeutic outcomes [53]. Notably, GITR expression was more pronounced and extended to a larger cell fraction on different immune cell subtypes in mice receiving the combinatorial treatment (Fig. 6B). This may be explained by a feedback loop mechanism involving GITR signaling activation and the upregulation of GITR expression through the NF-kappaB pathway [54]. Furthermore, the rapid tumor growth in our animal models may have outpaced the time needed for immune cells to activate and infiltrate the tumor, limiting the therapeutic window for interventions like TGF-β inhibition and GITR agonism [55]. This aligns with findings from other studies showing that the speed of tumor growth in preclinical glioma models correlates with poor responses to immunotherapies, especially those requiring immune cell activation and infiltration [56]. GITR modulation combined with immune checkpoint inhibition may require time to generate a robust immune response. Combined GITR and PD-1 targeting resulted in superior therapeutic activity in preclinical tumor models compared to either treatment alone [57]. A limitation of our study is the use of mouse glioma models. While these syngeneic models are valuable for mechanistic and therapeutic investigations [58], they only partially reflect the complexity of human glioblastoma, including its infiltrative growth, genetic heterogeneity, and immunosuppressive microenvironment. Compared to PD-1, TGF-β may represent an even more attractive combination partner for the therapeutic targeting of gliomas, given its central role in the biology of these tumors. Moreover, early clinical trials have already shown promising results using GITR agonists to modulate the immunosuppressive tumor microenvironment when combined with conventional therapies or PD-1 inhibitors in patients with solid tumors, including glioblastoma [59–61]. The findings of our study highlight the therapeutic potential of combining GITR and TGF-β pathway modulation and support further investigation of such combinatorial approaches for glioblastoma treatment.

## Supplementary Information

Below is the link to the electronic supplementary material.

Supplementary file1 (DOCX 20 KB)

Supplementary file2 (JPG 433 KB)

Supplementary file3 (JPG 219 KB)

Supplementary file4 (JPG 244 KB)

Supplementary file5 (DOCX 19 KB)

## Data Availability

The data that support the findings of this study are available from the corresponding author upon reasonable request.
