# Supplementary material for: Simultaneous TGF-β and GITR pathway modulation promotes anti-tumor immunity in glioma
Source: Cancer Immunol Immunother. 2025 Jun 28;74(8):254. doi: 10.1007/s00262-025-04098-w (PMC12206220; doi:10.1007/s00262-025-04098-w)
Supplement: Supplementary file 1 — Supplementary file1 (DOCX 20 KB) [file 262_2025_4098_MOESM1_ESM.docx]

**Supplementary Figure legends**

**Figure S1. Expression of immune checkpoint ligands in mouse glioma cell lines.** A-C. SMA-560, SMA-540, CT2A or GL-261 glioma cells were exposed to hr-TGF-β_2_ (10 ng/ml) or SD208 (1 μM) for 24h. The expression levels of PD-L1, CD137L, OX40L and LAG3 (A) or GITRL (B) were assessed by qPCR. C. Mouse glioma cells were treated as in (A) and GITRL protein expression was assessed by flow cytometry.

**Figure S2. GITR expression on immune cells.** A. GITR median fluorescence intensity in splenocytes treated with hr-TGF-β_2_ (10 ng/ml), SD208 (1 μM) or left untreated for 48 h. Data are expressed as mean ± SD. Statistical significance was assessed by one-way ANOVA with Dunnett’s post hoc test for multiple comparisons (* p<0.05; ** p<0.01; *** p<0.001). B. GITR protein levels at the cell surface were analyzed by flow cytometry in spleen-derived CD3^+^ T cells (upper panels) and CD3^-^ NKp46^+^ NK cells (lower panels).

**Figure S3. Characterization of GITR expression in the tumor microenvironment of syngeneic SMA-560 mouse gliomas.** A. Tumor-infiltrating immune cells were isolated and GITR protein levels at the cell surface were examined in NK cells (NKp46^+^ cells), myeloid cells (CD11b^+^ cells) as well as CD4^+^ and CD8^+^ T cells by flow cytometry. B. Twenty thousand SMA-560 cells were intracranially inoculated into the right striatum of syngeneic VM/Dk mice. From day 5 onward, mice were treated with a TGF-βRI inhibitor (LY2157299, 150 mg/kg, daily oral gavage), an agonistic GITR antibody (3 mg/kg, i.p. at days 5, 7 and 9), or a combination thereof. An untreated (control) group was included. The untreated and LY2157299-treated groups correspond to the cohorts in Fig. 5D. Survival data are shown, including the number of long-term surviving mice.
