# Supplementary figures and images for: Simultaneous TGF-β and GITR pathway modulation promotes anti-tumor immunity in glioma
Source: Cancer Immunol Immunother. 2025 Jun 28;74(8):254. doi: 10.1007/s00262-025-04098-w (PMC12206220; doi:10.1007/s00262-025-04098-w)

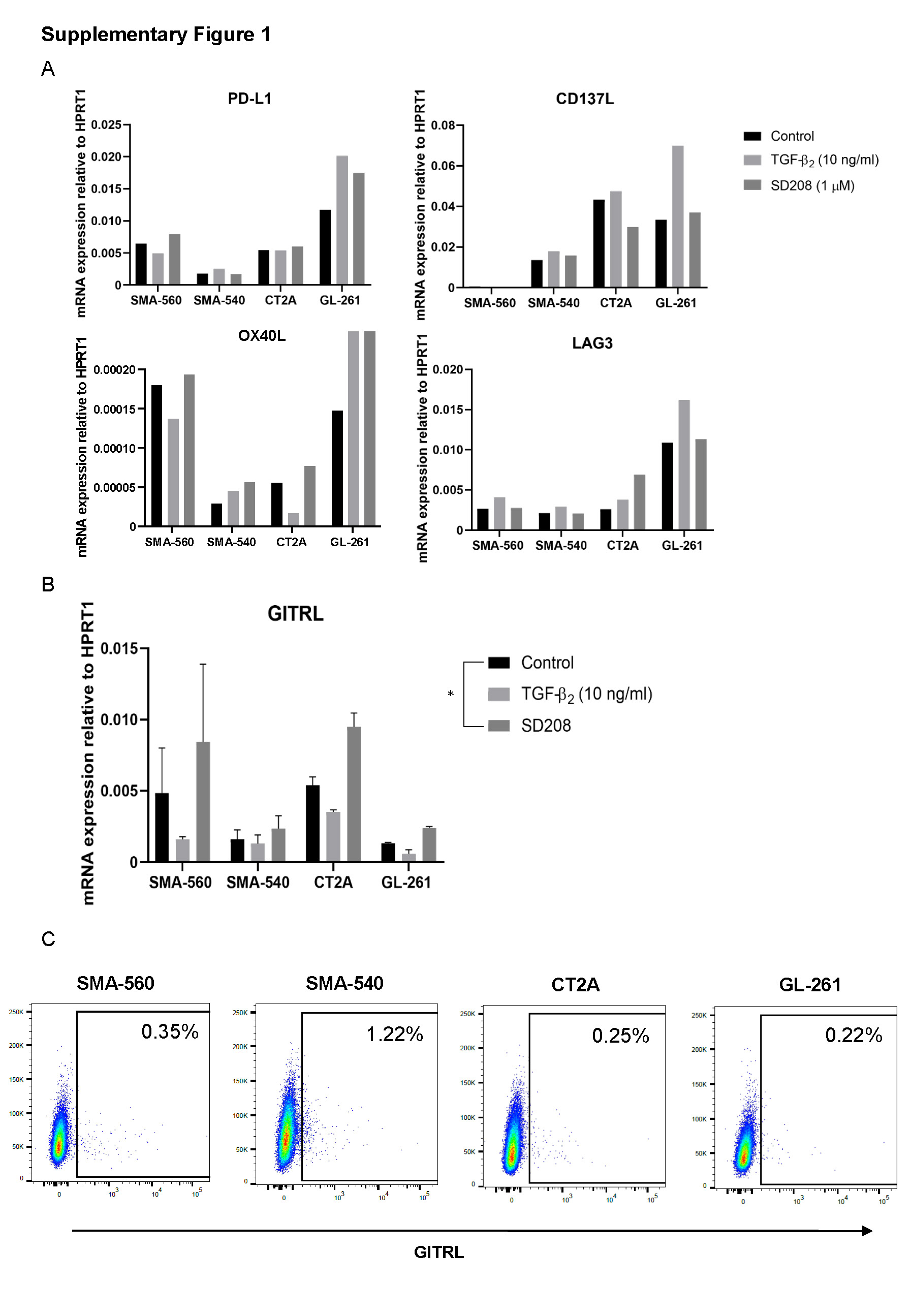

Supplement: Supplementary file 2 — Supplementary file2 (JPG 433 KB) [file 262_2025_4098_MOESM2_ESM.jpg]

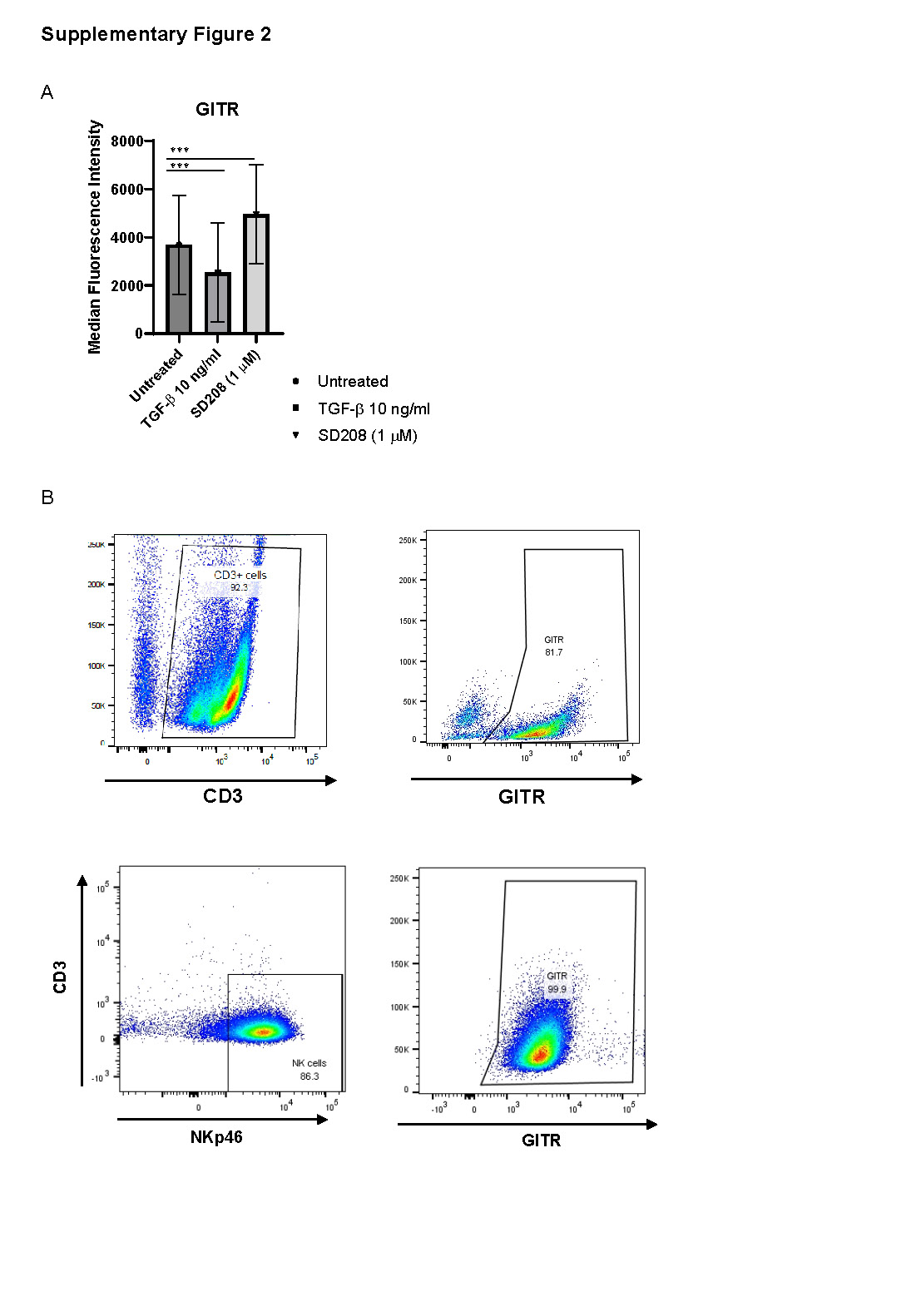

Supplement: Supplementary file 3 — Supplementary file3 (JPG 219 KB) [file 262_2025_4098_MOESM3_ESM.jpg]

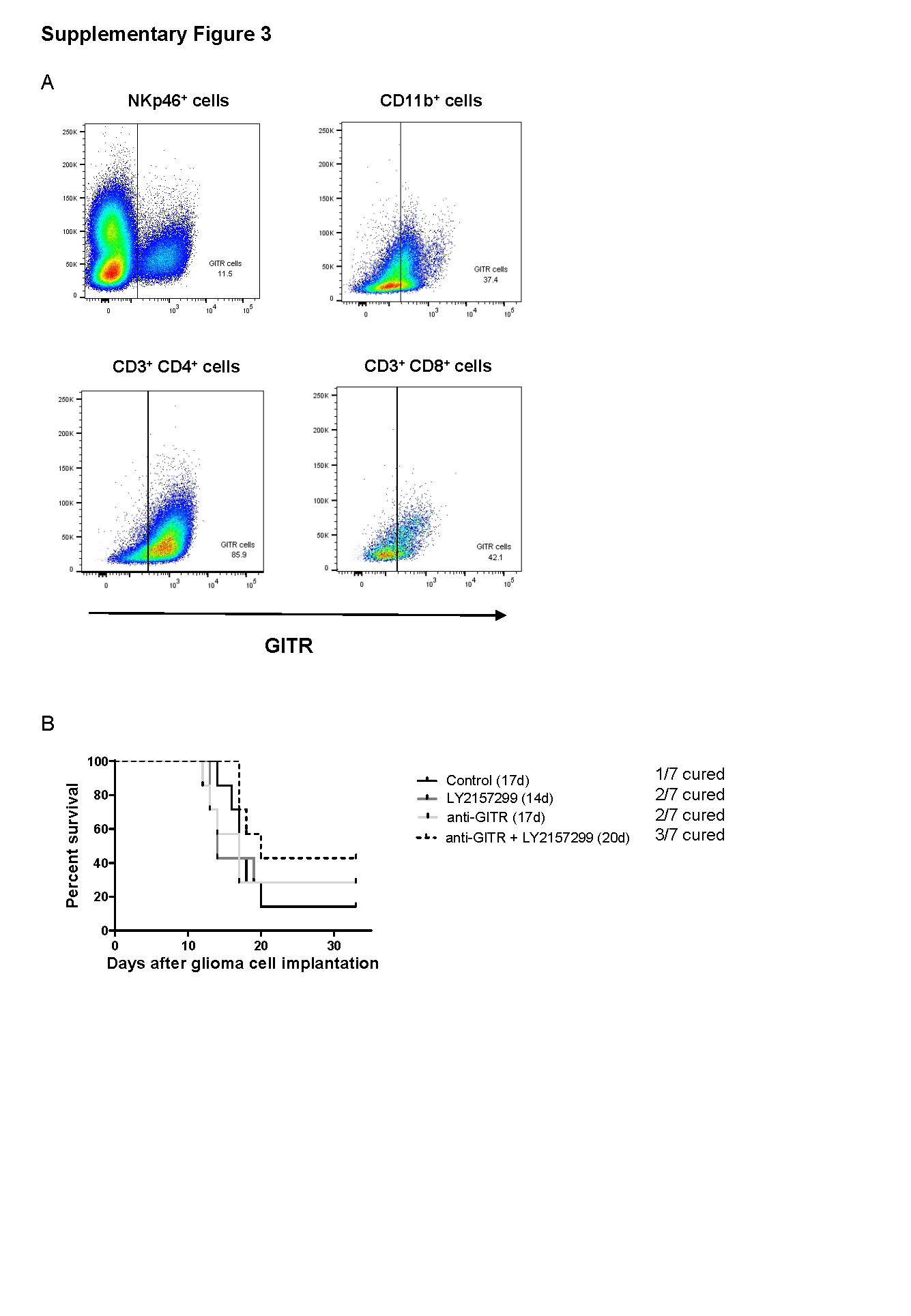

Supplement: Supplementary file 4 — Supplementary file4 (JPG 244 KB) [file 262_2025_4098_MOESM4_ESM.jpg]
