# Supplementary material for: Simultaneous TGF-β and GITR pathway modulation promotes anti-tumor immunity in glioma
Source: Cancer Immunol Immunother. 2025 Jun 28;74(8):254. doi: 10.1007/s00262-025-04098-w (PMC12206220; doi:10.1007/s00262-025-04098-w)
Supplement: Supplementary file 5 — Supplementary file5 (DOCX 19 KB) [file 262_2025_4098_MOESM5_ESM.docx]

**Suppl. Table 1**

| **Immune checkpoint receptor** | **Synonym** | **Cellular expression** | **Ligand** |
| --- | --- | --- | --- |
| PD-1  (immunosuppressive checkpoint) | CD279 | Activated T cells in peripheral tissue, B cells, professional APC, NK cells | PD-L1 (B7-H1)  (and PD-L2/ CD273) |
| LAG3  (immunosuppressive checkpoint) | CD223 | Activated T cells, NK cells | MHC class II |
| CD137  (immune-activating checkpoint) | 4-1BB | Activated CD4^+^ and CD8^+^ T cells, Tregs, activated NK cells, DCs, neutrophils | CD137L (4-1BBL) |
| GITR  (immune-activating checkpoint) | CD357 | Tregs, activated CD4^+^ and CD8^+^ T cells | GITRL |
| OX40  (immune-activating checkpoint) | CD134 | Activated CD4^+^ and CD8^+^ T cells, NK cells, neutrophils | OX40L |

Suppl. Table 1. Panel of investigated immune checkpoint molecules.
